# SCOBY-based, innovative, and sustainable production of gallic acid from sucrose towards multipurpose applications

**DOI:** 10.1038/s41598-025-24371-3

**Published:** 2025-11-18

**Authors:** Sonia Medina, Concepción Medrano-Padial, Silvia Guillén, Laura Pérez-Través, Irene Pérez-Novas, Paula Periago, Cristina García-Viguera, Raúl Domínguez-Perles

**Affiliations:** 1 Laboratorio de Fitoquímica y Alimentos Saludables (LabFAS), CSIC, CEBAS, Campus Universitario de Espinardo, Edificio 25, Murcia, 30100 Spain; 2 Associated Unit of R&D and Innovation CSIC-CEBAS+UPCT on “Quality and Risk Assessment of Foods”, Murcia, Spain; 3 Agronomic Engineering Department, Universidad Politécnica de Cartagena (UPCT), Paseo Alfonso XIII, 48, Cartagena, 30203 Spain; 4 Departamento de Producción Animal y Ciencia de los Alimentos, Instituto Agroalimentario de Aragón - IA2 - (Universidad de Zaragoza-CITA), Zaragoza, Spain; 5 Insituto de Agroquímica y Tecnología de Alimentos, IATA, CSIC, Catedrático Agustín Escardino Benlloch, 7, Paterna, 46980 Valencia Spain

**Keywords:** Sucrose, Microbial consortium, Natural source, Biosynthesis, Gallic acid, Biochemistry, Biotechnology, Chemical biology

## Abstract

Kombucha is a traditional beverage obtained from the fermentation of sugared tea by a symbiotic culture of bacteria and yeast (SCOBY), whose metabolism contributes significantly to the phytochemical composition and health-promoting properties of the final product. Among the phenolics present, gallic acid stands out as a multifunctional molecule with antioxidant, anti-inflammatory, and cardio-protective activities, making it a compound of growing interest for the development of functional foods, nutraceuticals and cosmetics. While gallic acid in kombucha has typically been attributed to plant-derived precursors, its potential *de novo* microbial origin has remained largely unexplored. In this work, robust evidence supports that SCOBY can synthesise gallic acid directly from sugars, without the contribution of tea or other plant materials. Metabolomic analyses combined with physicochemical characterisation (pH, ethanol, acetic acid, total soluble solids, sucrose, glucose, and fructose) revealed a linear increase in gallic acid production under standard fermentation conditions, associated with the microbial community’s tolerance to high sugar concentrations and its metabolic capacity to generate bioactive phenolics. This finding highlights a previously unrecognised role of SCOBY as a natural cell factory for gallic acid production. In contrast to metabolic engineering approaches in model microorganisms such as Escherichia coli or Pseudomonas, our study demonstrates that a non-engineered microbial consortium can achieve this transformation simply and sustainably. These results open a novel route for the plant-free biosynthesis of gallic acid with potential applications across the food, cosmetic, and pharmaceutical industries.

**Supplementary Information:**

The online version contains supplementary material available at 10.1038/s41598-025-24371-3.

## Introduction

In recent years, symbiotic cultures of bacteria and yeasts (SCOBY) have garnered considerable interest from both the scientific community and the beverage industry as sources of biodegradable and sustainable multipurpose materials. SCOBY’s primary role has traditionally involved fermentation of plant-based food substrates (e.g., green or black tea) to produce kombucha, whose market is expanding rapidly to achieve approximately USD 4.26 billion in 2024, projected to reach USD 9.09 billion by 2030 (Compound Annual Growth Rate of almost 14.0% approximately) according to recent market reports (Grand View Research, 2024^1^. In addition, SCOBY forms a cellulose-based biofilm at the air-liquid interface during fermentation^2^. However, in the course of this process, the microbiota in SCOBY not only synthesises cellulose but also metabolically transforms tea-derived compounds^3^, contributing to the beverage’s phenolic composition and health-promoting attributes^4^. The probiotic properties and the phytochemical profile resulting from the SCOBY metabolism are therefore key to the functional quality of the beverage^5^. Accordingly, the phytochemical burden of kombucha strongly depends on the microbial composition of the consortia and their ability to metabolise plant-derived compounds into new bioactive molecules^6^. However, whether SCOBY can also generate phenolic compounds, such as gallic acid, *de novo* in the presence of plant precursors remains largely unexplored.

Among the phenolics present in kombucha, gallic acid is especially notable due to its antioxidant, anti-inflammatory, and anticancer activities. These properties convert this phenolic into a polyvalent compound of interest for food, pharmaceutical, and cosmetic industries^7^. As a result of these functional traits, the global gallic acid market is projected to reach US$131.1 million by 2030^8^.

Although most gallic acid in fermented beverages originates from plant material, hydrolysis and degallation reactions occurring during fermentation may also give rise to newly formed gallic acid^6^. Nonetheless, these routes do not fully explain the total amount of gallic acid detected in fermented beverages, and alternative microbial or physicochemical pathways have been hypothesised. One such route involves the conversion of glucose (or other sugars) to gallic acid through a microbe-catalysed process in the shikimate pathway, where intermediates such as 3-dehydroshikimic acid are dehydrated to protocatechuic acid^9^ and subsequently hydroxylated to gallic acid by specific microbial enzymes, including 3-dehydroshikimic acid dehydratase (e.g., from *Klebsiella pneumoniae*)^9^ and *p*-hydroxybenzoate hydroxylase (e.g., from *Pseudomonas aeruginosa*)^10,11^. Moreover, metabolically engineered systems (e.g., recombinant *Escherichia coli*) have achieved gallic acid production directly from glucose^11,12^.

Despite this body of evidence on the metabolic capacity of new metabolically engineered systems, no studies have demonstrated that SCOBY, in the absence of plant-derived precursors, can autonomously produce gallic acid from simple sugars such as glucose or fructose. This represents a critical knowledge gap that differentiates this work from previous kombucha research (which almost invariably relies on plant-based substrates), as well as from metabolic engineering approaches in model organisms like *E. coli* or *Pseudomonas*. These approaches rely on genetically modified monocultures optimised for high concentrations under controlled conditions^12,13^. In contrast, demonstrating that a naturally occurring microbial consortium can biosynthesise gallic acid without genetic manipulation or complex process design would highlight the intrinsic metabolic versatility of SCOBY and point it out it as an intermediate alternative between traditional fermentation practices and advanced biotechnological systems.

Hence, this study situates SCOBY fermentation at the intersection of traditional kombucha fermentation and synthetic biology. This approach opens a novel route for the sustainable, plant-free biosynthesis of a high-value phenolic compound that may serve as an eco-friendly alternative or complement to existing microbial and plant-based production strategies. To this end, metabolomic approaches, proximate analysis, and microbiological profiling were applied. The joint analysis of this data uncovers the microbe-mediated production of natural gallic acid from glucose, in close agreement with metabolic traits of the microbial species integrating the SCOBY consortium.

## Results and discussion

**Fig. 1:**
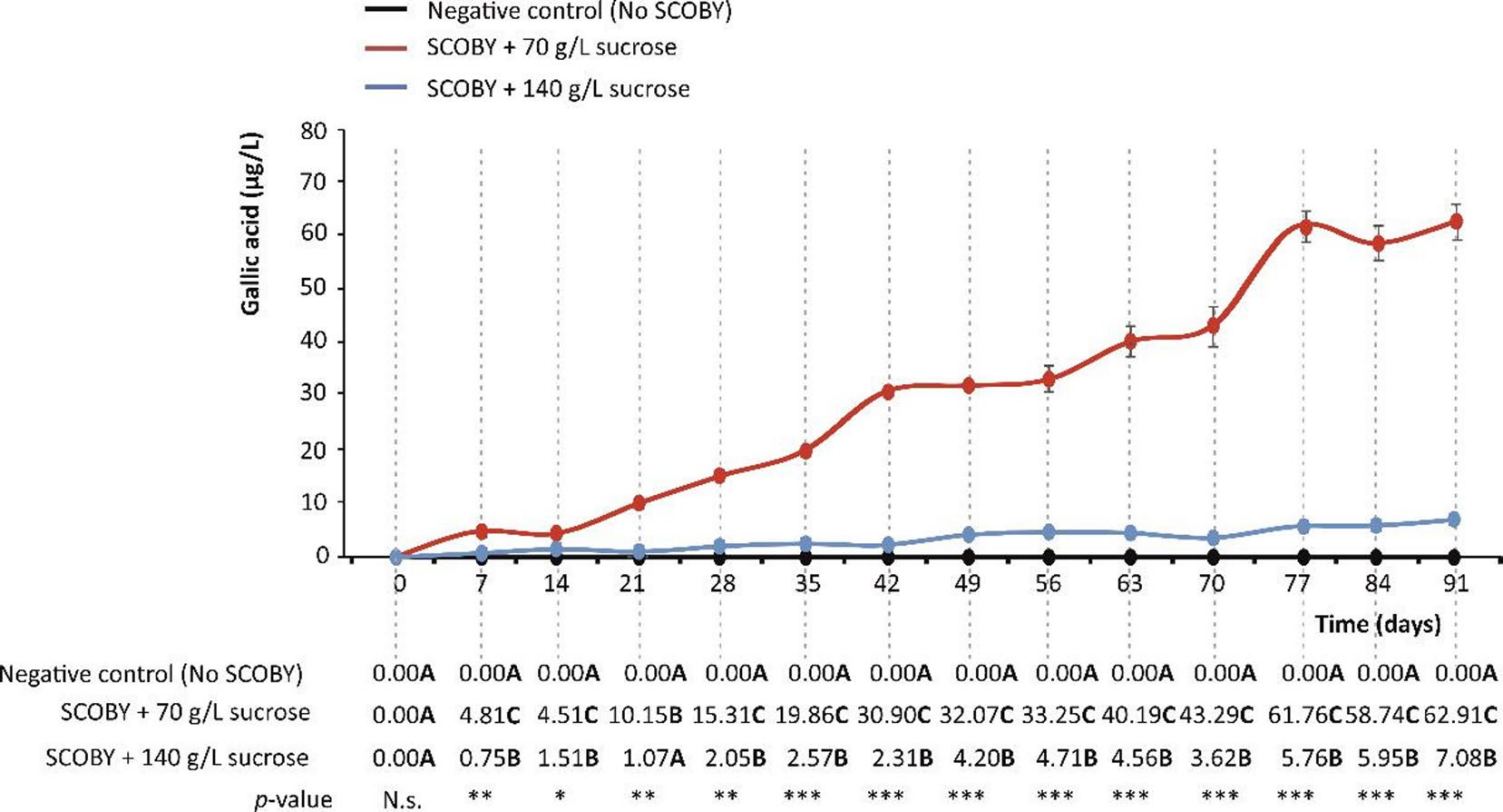
Gallic acid concentration (µg/L) in the symbiotic culture of bacteria and yeast (SCOBY) growing media supplemented with 70 and 140 g/L of sucrose in 91 days of incubation at room temperature, protected from light. Distinct bold capital letters indicate concentrations of gallic acid are significantly different according to one-way analysis of variance (ANOVA) and multiple range test of Tukey (*n* = 3). N.s., not significant; *p* < 0.05*; *p* < 0.01**; *p* < 0.001***.

**Fig. 2:**
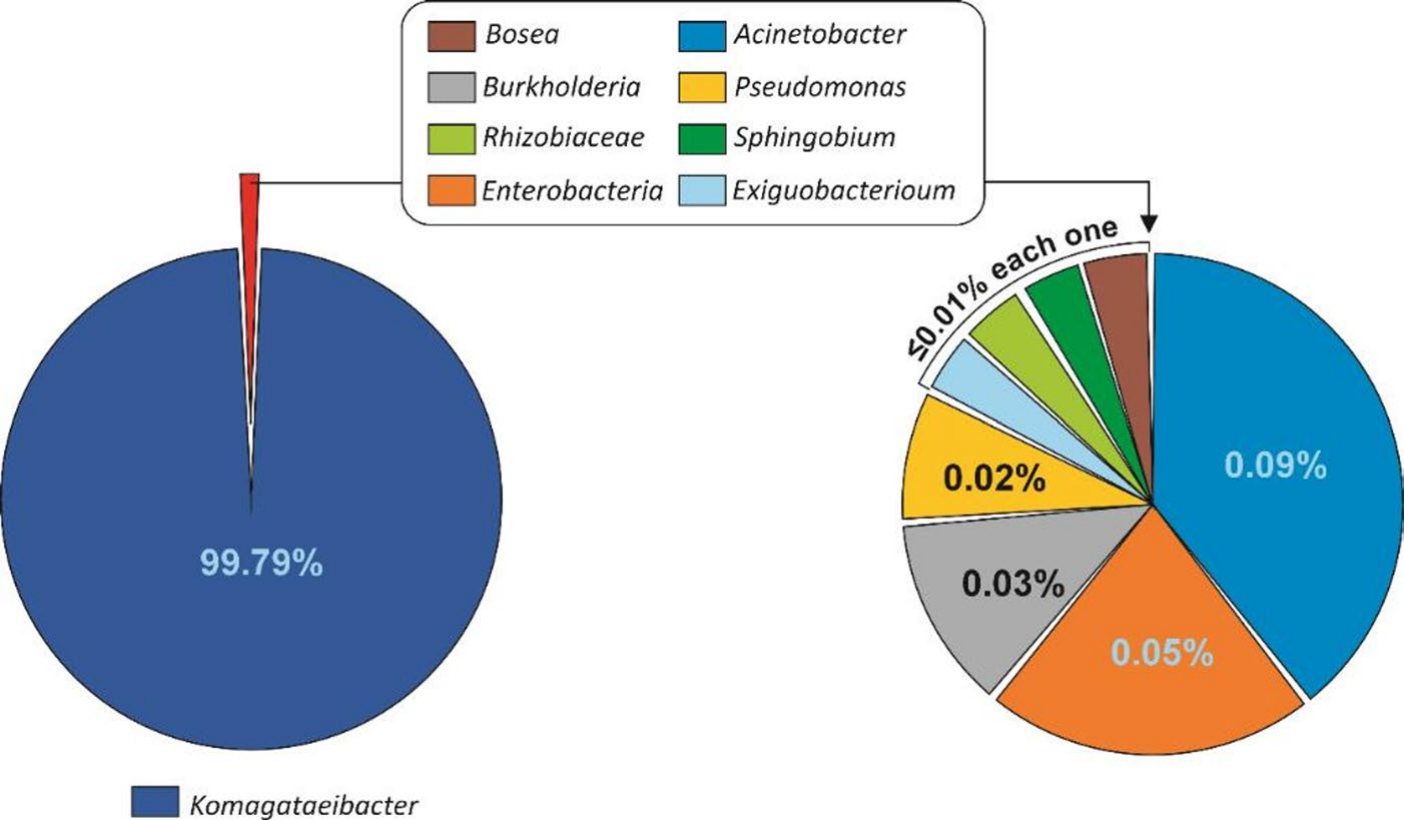
Diversity of bacteria in the symbiotic culture of bacteria and yeast (SCOBY) according to the microbiological identification in the 14-day-old microbial suspension.

**Fig. 3:**
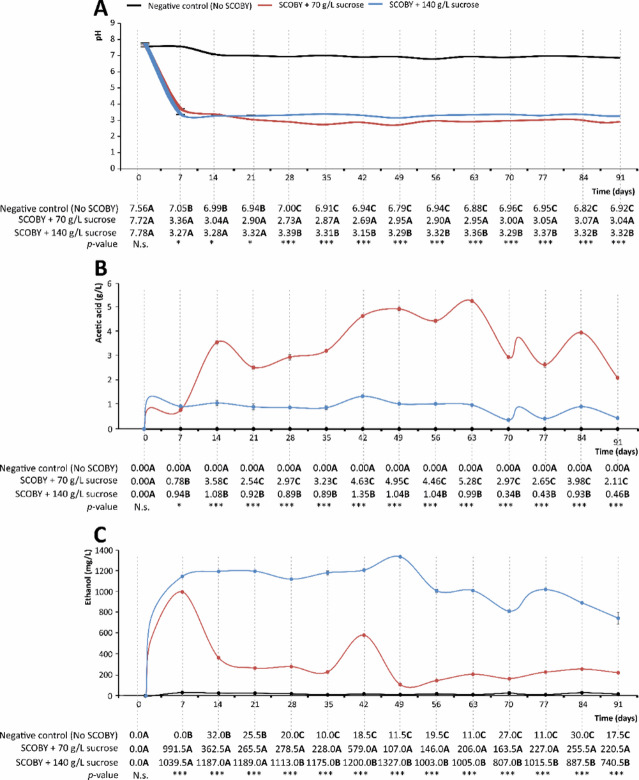
A pH (**A**), acetic acid (g/L) (**B**), and ethanol (mg/L) (B) in the symbiotic culture of bacteria and yeast (SCOBY) growing media supplemented with 70 and 140 g/L of sucrose in 91 days of incubation at room temperature, protected from light. Distinct bold capital letters indicate concentrations of gallic acid are significantly different according to one-way analysis of variance (ANOVA) and multiple range test of Tukey (*n* = 3). N.s., not significant; *p* < 0.05* and *p* < 0.001***.

**Fig. 4:**
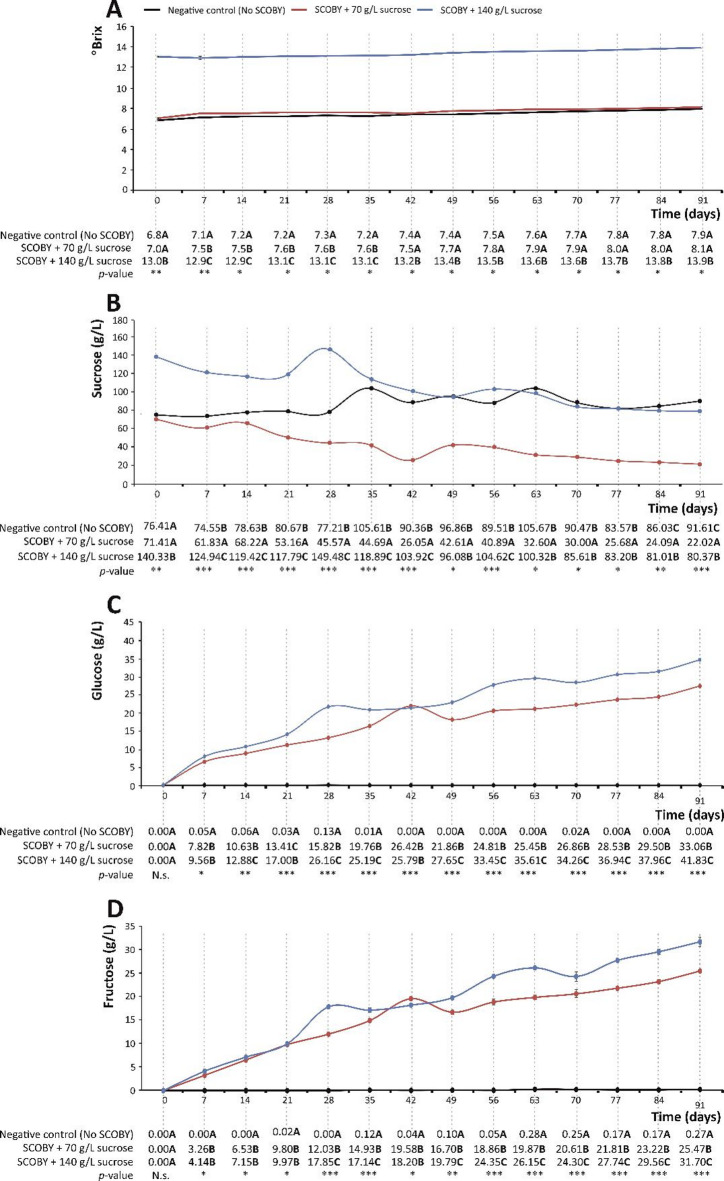
Total soluble solids (TSS) (**A**), sucrose (g/L) (**B**), glucose (g/L) (**C**), and fructose (g/L) (**D**) in the symbiotic culture of bacteria and yeast (SCOBY) growing media supplemented with 70 and 140 g/L of sucrose in 91 days of incubation at room temperature, protected from light. Distinct bold capital letters indicate concentrations of gallic acid are significantly different according to one-way analysis of variance (ANOVA) and multiple range test of Tukey (*n* = 3). N.s., not significant; *p* < 0.05*; *p* < 0.01**; *p* < 0.001***.

The growing demand for healthy foods and beverages has boosted the consumption of fermented products. To further support this rising consumption and unravel the real effects on health, the phytochemical profile and probiotic properties attributed to fermented drinks have been comprehensively characterised^14^. Hence, beyond the phytochemicals provided by the plant material and closely dependent on the metabolic traits of bacteria and yeasts making part of the SCOBY, the original phytochemical profile undergoes modifications^15^, endowing fermented beverages with a range of postbiotic derivatives present at operative concentrations. This fact would give rise to a qualitative difference relative to the unfermented matrix or the original plant material, while being associated with enhanced biological attributes^16^. However, the influence of phenolic compounds on microorganisms’ metabolism is complex^17^, and the lack of studies on the microbial species responsible for the phytochemical profile of kombuchas, especially regarding the formation of gallic acid, prompted us to evaluate the capacity of the microbial consortia to synthesise this bioactive phenolic from sucrose, in the absence of plant material. To facilitate comparison, key fermentation parameters, gallic acid levels, and microbial profile are summarised in Table S1, while full time-course assessments are depicted in Figs. 1, 2 and 3, and 4.

### Gallic acid concentration in the SCOBY-sugared media

**Fig. 5:**
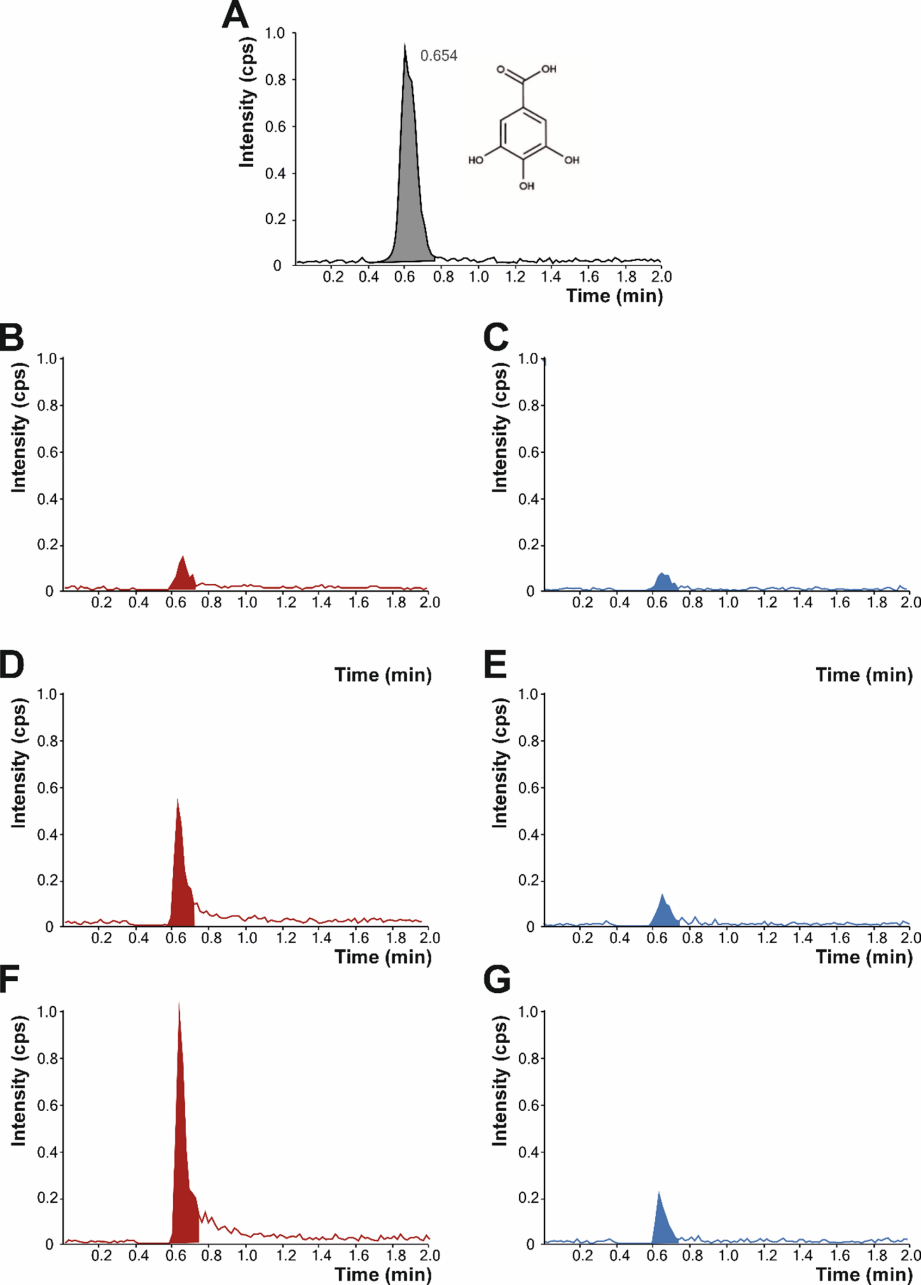
Representative ultra-high performance liquied chromatography coupled an electrospray ionisation systema an triple-quadrupole-mass spectrometer (UHPLC-ESI-QqQ-MS/MS) chromatograms of 1 µM gallic acid (authentic standard) (A), and the phenolic acid normalised by the symbiotic culture of bacteria and yeast (SCOBY) growth at 70 and 140 g/L of sucrose at days 7 (B and C, respectively), 49 (D and E, respectively), and 91 (F and G, respectively). The scale was normalised to 1 × 10^4^ cps to represent the relative abundance under the different production conditions (sucrose concentration and time).

When analysing the presence of gallic acid in the sugared solutions, the formation of gallic acid was not observed in the negative control (no SCOBY) (Fig. 1). This fact supports the essential participation of the array of microbial genera in the medium for the synthesis of gallic acid, according to their specific metabolic traits. However, significant differences in the formation of gallic acid were observed depending on the amount of sucrose added to the solutions (70 and 140 g/L). Thus, 70 g sucrose/L allowed the SCOBY to produce gallic acid almost linearly (R^2^ = 0.9721) up to 62.91 µg/L, after 91-day fermentation. Under these conditions, the gallic acid yield per gram of sugar consumed at 91 days, as a result of the SCOBY metabolism, was 1.28 µg. It may be observed that the degradation of sucrose (at 70 g/L) by the SCOBY had a good correspondence with a first-order kinetic model (Fig. S1), displaying a rate constant (*k*) for the degradation process of 0.011 day^–1^ and a conversion rate to gallic acid of 63.2% at 91 days (Figure S1). This low value for *k* indicates a sluggish consumption of sucrose^18^ that would achieve ~ 50% o the initial concentration the day 63, which was confirmed by the experimental data (Figure S1). Interestingly, the experimental results recorded for 70 g/L differed significantly from the 7.08 µg/L produced (also linearly, R^2^ = 0.9294) at 140 g sucrose/L, showing nearly nine times lower concentration (Figs. 1 and 5). Anyway, during the process, the degradation ratio would depend on the amount of substrate available at each time-point within the operative range for the different factors (e.g., temperature, time, or pH, among others) involved in the productive system, to achieve an eventual industrial scaled-up, shorting fermentation time and thus, enhancing the industrial feseability towards more robust and sustainable settings, as evidenced by the results retrieved for 140 g sucrose/L.

Considering the standard temperature in this research helped us to restrict the eventual factors responsible for modifying microbial metabolism^15^, allowing us to reach more robust conclusions. Thus, the fermentation temperature chosen was based on the requirements for maintaining bacteria and yeast metabolisms^19^. Accordingly, the production temperature was standardised in the range of 24–30 °C to achieve the optimum synthesis of bioactive molecules^19^. In this connection, most results obtained suggested that (poly)phenols formed during fermentation would depend on two main factors. First, the microbial composition of the SCOBY (and their characteristic metabolic traits) and secondly, the starting chemical profile in the fermented beverage’s matrix, represented in our experimental design by the presence/absence of sucrose and its concentration^20^. Accordingly, beyond the specific metabolic traits characterising the diverse bacteria and yeast species in the SCOBY, the amount of gallic acid formed under different conditions would indicate that the efficiency of the process is severely associated with the sugar content. This fully agrees with the recent description of the relationship between the sugar levels and microbial metabolism. For instance, contents higher than 125 g/L may reduce the microbial metabolism concerning organic acids production due to the imbalance of nutritional uptake and utilisation^14^. Moreover, sugar concentrations > 125 g/L may induce osmotic stress on microorganisms, thus suppressing their growth^21^ and, consequently, the metabolic capacity to modify the (phyto)chemical profile during fermentation.

Time is an additional factor that should be considered critical based on previous evidence of the existing relationship with the radical scavenging power of beverages. Indeed, some chelating compounds responsible for the antioxidant profile of kombucha could be generated over time^22^.

Other key factors for the production of bioactive compounds in the matrix of fermented beverages are the age and composition of the SCOBY. Thus, younger SCOBY with fewer cycles of fermentation may have different metabolic activity compared to older microbial consortia. This has been attributed to the fact that consecutive cycles of fermentation of SCOBY might lead to a change in the microbial diversity and its metabolic capacity and, therefore, its potential to produce postbiotic bioactives^23^.

According to the results obtained from these previous studies, the influence of time and fermentation cycles on the production of gallic acid from sugar by microbial consortia requires further clarification. Interestingly, this association also works in the reverse way, since phenolic compounds (e.g., gallic acid) may influence the diversity and metabolic activity of the microbial consortium. Beyond this, possible limitations would include depletion of nitrogen, phosphorus, or micronutrients required for enzyme activity in the shikimate pathway, as well as reduced microbial viability under low pH and high organic acid conditions. Therefore, adjusting media composition (e.g., supplementation with nitrogen sources or mineral nutrients acting as key enzymes’ co-factors) could enhance yields and should be explored in future optimisation studies^24^.

### Bacteria and yeast diversity in the SCOBY

#### Bacterial diversity in the SCOBY

High-throughput microbial community sequencing revealed Firmicutes as the dominant bacterial Phylum (99.99%) and, to a minor extent, Proteobacteria (0.01%). Thus, the assessment of the microbial profile in the SCOBY revealed the presence of 9 different types of bacteria. *Komagateibacter* was the dominant one with an abundance of more than > 99.00%. Other bacteria identified were, in decreasing order of abundance, *Acinetobacter* > *Enterobacter > Burkholderia > Pseudomonas > Exiguobacterium*, *Rhizobiaceae*, *Sphingobium*, and *Bosea* (Fig. 2).

The microbial identification was consistent with previous research describing *Komagateibacter* as the preponderant species in the SCOBY^25^. This species is an acetic acid bacterium, responsible for producing acetic and glucuronic acids. In addition, *Komagataeibacter* develop a cellulose network. This capacity contributes to enhancing the association between bacteria and yeasts that form a thick, gelatinous-looking membrane, with its maximum bacterial cellulose production at 14 days^26^. Thus, the presence of bacteria from the *Komagataeibacter* genus conditions the development of the SCOBY^27^. Its relative abundance has been correlated with high glucose concentrations^28^, even though fructose seems to be preferentially utilised over glucose in the kombucha fermentation process^29^. Moreover, high *Komagataeibacter* abundance has been associated with the low pH featured in kombucha, resulting, to some extent, from the metabolic transformation of sugar into organic acids. This is consistent with a high tolerance of *Komagataeibacter* against acidic environments^30^.

Concerning the contribution of the separate bacteria identified in the SCOBY to the fermentative events, despite the low proportion of *Acinetobacter*, *Rhizobiaceae*, and *Sphingobium* species, they would provide significant inputs by producing volatile flavour substances or phenolic compounds^31^.

The presence of genera such as *Pseudomonas* or Enterobacteriaceae (in low numbers), which are considered indicator microorganisms with no risk at low concentrations, includes mostly non-pathogenic spp and some pathogenic ones, does not imply a health risk. Additionally, the nature of kombucha (with a pH below 4, temperature, and aeration conditions) would not support their growth; therefore, the product can be considered safe. Nonetheless, in some cases, they have been identified^32^.

#### Yeast diversity in the SCOBY

The colonies developed in glucose-peptone yeast media were classified into two different morphological categories, according to their phenotypic characteristics (colour). The molecular analyses supported the presence of *Zygosaccharomyces bailii* colonies as cream-coloured, while the salmon-coloured ones exhibited molecular traits matching *Rhodotorula mucilaginosa*.

According to previous reports, the yeast profile of the SCOBY is highly diverse and can vary in parallel with changes in environmental conditions^29^. Thus, traditionally, the dominant and relevant yeast genera for Kombucha production are *Zygosaccharomyces*,* Candida*,* Torulaspora*,* Pichia*,* Brettanomyces*,* Schizosaccharomyces*,* Hanseniaspora*, and *Saccharomyces*. Moreover, to a lesser extent, *Rhodotorula*, *Lachancea*, and *Starmerella* also participate in fermentations^33^. This evidence is in good agreement with the characterisation of the yeast diversity in the SCOBY described in the present work. Hence, *Zygosaccharomyces* has been more frequently described as the dominant yeast genus in Kombucha. Alternatively, *R. mucilaginosa* has also been described. This yeast profile contributes to further understanding and contextualising previous evidence in the literature, demonstrating the close relationship between the metabolic traits of particular yeast species and the production of bioactive compounds (e.g., carotenoids). This knowledge allows taking advantage of optimising media for microbial growth and fermentation^34^. These antecedents support the envisagement of production settings for specific key bioactive phenolics upon developing fermented foods^35^. Interestingly, this species (*R. mucilaginosa*) is enclosed at the beginning of the kombucha’s performance when unmetabolised glucose is available. Nonetheless, since the growth rate and metabolic activity have been associated with the physicochemical condition of the medium^34^, assessing the model systems developed in the present work is essential to understanding the relevance of the different genera to the overall gallic acid production.

To gain insights into the integrants of the microbial consortia with responsibility among the separate species and genera identified, the metabolic traits featuring the different components should be considered. Previous studies have described 3-dehydroshikimic acid dehydratase activity in bacteria (e.g., *K. pneumoniae*)^9^, but not in any of the spp. identified in our SCOBY (Fig. 2). In our study, the pathway for the synthesis of gallic acid was not established. Therefore, identifying the microbial species with the capacity to produce enzymes that synthesise gallic acid from glucose would be a chance to enhance the microbial consortium to obtain fermented products with an enriched phytochemical profile. Accordingly, identifying the microbial species with the capacity to produce the enzymes involved in synthesising gallic acid from glucose would be a chance to fine-tune the microbial consortium to obtain fermented products with an enriched phytochemical profile.

Despite the lack of gene annotation in the present work, the putative biosynthetic routes for gallic acid present in the microbial species identified in the SCOBY are in good agreement with the complementary bacterial metabolic mechanisms previously described and introduced in previous sections. These pathways could underlie the *de novo* formation of gallic acid from sucrose. Hence, the time-resolved accumulation of gallic acid in the absence of plant precursors strongly supports a microbial, shikimate-based origin consistent with these enzymatic routes.

### pH, the content of ethanol, and acetic acid in the SCOBY growing media

**Fig. 6:**
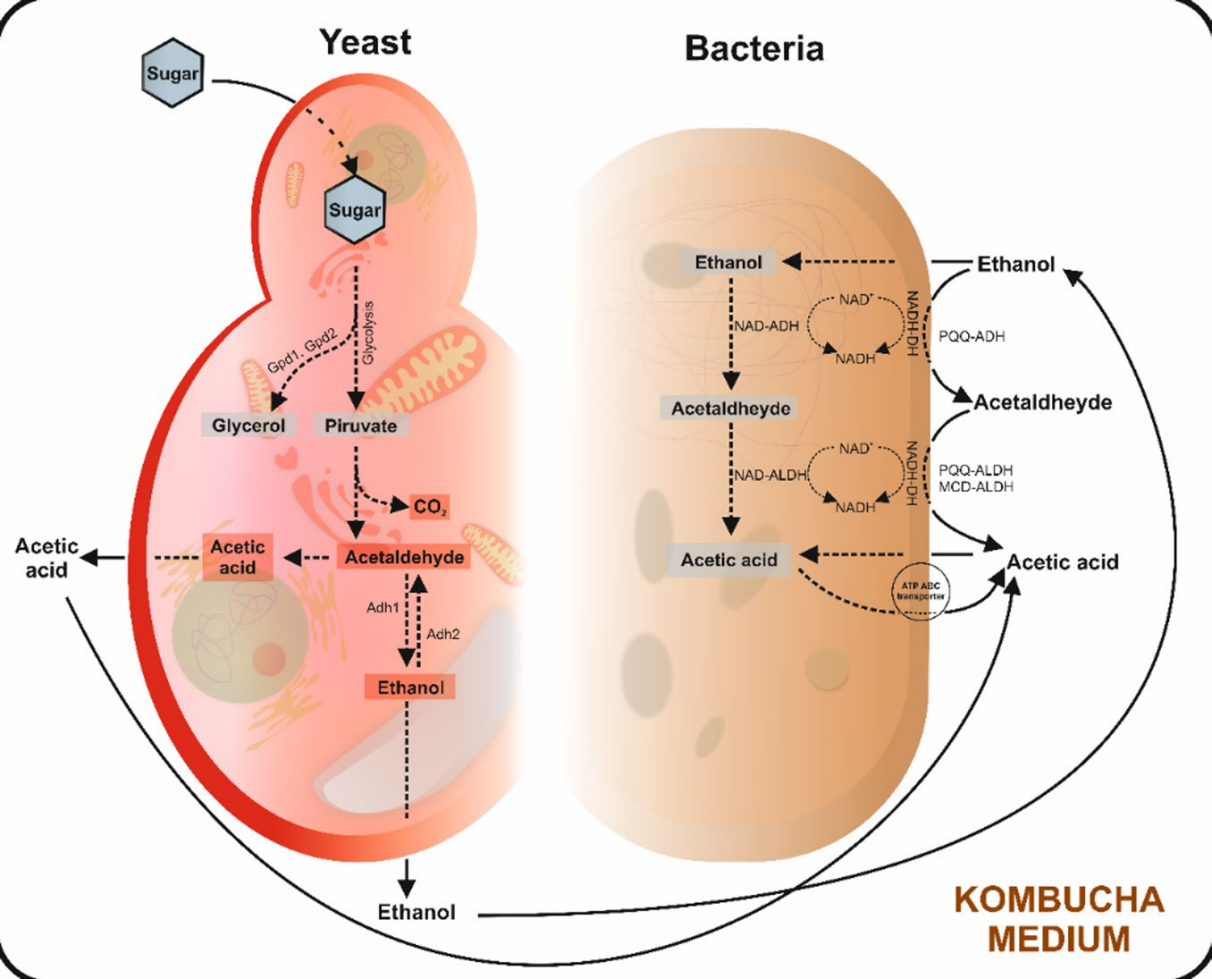
Microbial metabolism scheme.

As shown in Fig. 3, the initial pH for all models implemented (negative control, SCOBY + 70 g sucrose/L, and SCOBY + 140 g sucrose/L) was 7.69, on average. In the negative control, the pH slightly decreased during fermentation to achieve 6.92. The sucrose metabolisation upon SCOBY fermentation in 70 and 140 g sucrose/L solutions significantly caused the pH drop until day 7 (up to 3.72 and 3.36, respectively). Afterwards, the pH remained almost constant, reaching the final values of 3.04 and 3.32, respectively (Fig. 3A). This pH decrease agrees with previous descriptions^36^ that attributed it to the metabolic reactions responsible for producing organic acids, mainly acetic acid, due to sugar metabolisation by yeasts and bacteria (Fig. 6)^37^. Thus, maintaining pH within the referred values, based on the organic acid produced by the range of microbial components^38^, could contribute to preserving the chemical structure and biological activity of phytochemicals^24^. In this aspect, the pH induced by organic acids constitutes a selective criterion for specific bacteria and yeast^34^. As a result, the given consortium is featured by metabolic traits powerful enough to enrich the phytochemical profile, for instance, by displaying the enzymes required to synthesise gallic acid^39^. Previous assessments of kombucha-like models on quantitative organic acids evidenced that acetic acid could make up 40% of the toal^40^. To confirm the central role of acetic acid in pH lowering, changes in its concentration were monitored (Fig. 3B).

The amount of acetic acid in the negative control remained at a level lower than the limit of quantification (< LOQ) for 91 days. Alternatively, in the fermentation models (70 and 140 g sucrose/L), its concentration increased significantly up to 2.11 and 0.46 g/L, respectively. The differences recorded concerning the acetic acid content of SCOBY-like models could be related to the harmful effect of a high glucose environment (140 g/L) on the microbial metabolism^41^. Moreover, acetic acid concentration above 5 g/L can significantly inhibit microbial growth and metabolic activity^42^. When analysing the evolution of acetic acid over time, the length of the fermentation period seems not to be the only factor responsible for its synthesis (Fig. 3B). Temperature has been closely defined in the fermentation process (25 ± 2 °C) since it has been identified as a critical parameter that modulates the fermentation efficiency and the synthesis of organic acids. Most acetic acid bacteria (AAB) thrive at an optimal growth temperature of around 25–30 °C^43^. Hence, the concentration of (poly)phenols in black tea increases during 21-day fermentation at 20 °C, while at 30 °C the evolution is contrary^14^.

Beyond temperature, sugar is required as a carbon source during fermentation. Therefore, its concentration influences the production of organic acids, phenolic burden, and antioxidant activity of fermented beverages^14^. Again, all these factors should be considered to select specific microbial genera and species, which would allow obtaining a specific phytochemical profile and health attributes identified to date for fermented foods.

The organic acids in the media act as yeast stimulants to produce ethanol that AAB use to produce acetic acid (Fig. 6)^44^. Thus, the assessment of the fermented beverage models developed with 70 and 140 g sucrose/L suggested a rapid increase of the ethanol concentration during the first 7 days of fermentation (Fig. 3C), when the pH drop exhibited the highest slope (Fig. 3A and C). After day 7, the 70 and 140 g sucrose/L matrices exhibited significantly different ethanol content (401 and 1190 mg/L, on average, respectively) that remained almost constant until day 50. Then, ethanol decreased by 48.6%and 22.7% on average, in the matrices developed with 70 and 140 g sucrose/L, correspondingly (Fig. 3C). The differences between sucrose concentration and the 2-phase drop of ethanol agree with previous associations of such an event with the availability of sugars and the length of fermentation^45^. These changes are associated with the specific metabolism of the different microbial species and genera, and the collaborative role of yeasts that convert sucrose into ethanol and AAB that oxidise ethanol^46^. Beyond this, the metabolic activity of bacteria making part of the SCOBY converts ethanol into acetic acid, thus lowering the ethanol formation^47^.

### Evolution of sugar content upon fermentation

The development of kombucha beverages involves using sugars as a carbon source to complete the metabolic processes of the SCOBY^48^. Yeasts break sucrose, resorting to invertase and fermentative activity, providing glucose and fructose, which are converted into ethanol and carbon dioxide during fermentation (Fig. 6)^49^. Acetic acid bacteria use yeast metabolites to produce organic acids (e.g., acetic and gluconic acids), thus acidifying the matrix^49^ and further selecting the microbial and metabolic profile of the system^34,39^. Accordingly, the type and amount of sugar, as well as the fermentation time and temperature, influence the organic acids and phenolic content^14^, being required for the identification of the genera and species responsible for synthesising phytochemicals. Indeed, this information would be of interest for designing and obtaining gallic acid production biofactories.

In this concern, the type (sucrose) and concentration range (from 10 to 100 g/L) of the sugar applied for fermented drinks for commercial purposes^14^ have been the reference for the development of model systems unravelled in this study (70 g/L (7% *w/v*) and 140 g/L (14% *w/v*)). The fermentation temperature (25 ± 2 °C) was also in the range accepted for kombucha production, 24–30 °C^19^. Under these conditions, the evolution of sugar content was measured as TSS (Fig. 4A) and as sucrose, glucose, and fructose concentrations (Fig. 4B and D). This allowed gathering information on the day-by-day fermentation kinetics that, nowadays, remain scarcely documented. For 70 and 140 g sucrose/L, the evolution of this dimeric sugar followed an almost linear trend, decreasing by 69.2% ad 42.7%, espectively. This evolution supported the efficiency of the fermentation process. Additionally, the simultaneous augmentation of gallic acid concentration in the media could be attributed to the SCOBY microbial profile and its metabolic activity. The role of each microorganism in the metabolism leading to this activity should be studied further to establish the pathway responsible for gallic acid synthesis.

Concerning monomeric sugars (Fig. 4C and D), no glucose and fructose were detected in the control, whilst both of them experienced similar augmentations during fermentation up to 33.06 and 41.83 g glucose/L (Fig. 4C) and 24.47 and 31.70 g fructose/L (Fig. 4D) in the SCOBY media prepared at 70 and 140 g sucrose/L, respectively. The hydrolysis of sucrose towards two constituent monosaccharides is showcased by the periplasmic yeast invertase and is used by bacteria to produce gluconic acid, ethanol, and acetic acid upon oxidative metabolism^50^. Also, during the SCOBY growth, cellulose leads to the formation of the new pellicle, which can be used as an inoculum of the SCOBY in further fermentation^51^. This matrix entraps the microbial diversity and enables trophic and metabolic interactions among microbial communities^52^. The formation of a cellulosic pellicle, together with pH drops, might protect against contamination by exogenous spoilage agents^53^, thus ensuring that the fermentation process runs adequately.

Although this study demonstrates the ability of SCOBY to synthesise gallic acid *de novo* from sucrose, some limitations are worth stressing. The microbiological profile of the SCOBY has been analysed in the present work. However, the specific microbial species and enzymes responsible for this transformation remain unidentified, and the influence of fermentation cycles or environmental conditions on metabolic performance still requires further clarification. In agreement with this knowledge gap, future research should aim to isolate and functionally characterise the key microorganisms. In addition, annotating genes involved in the metabolic pathways critical for transforming molecules from the media, as well as microbiome diversity indices, evaluating scalability, and process optimisation for industrial purposes, would also be of great interest. Altogether, this information would lead to the rational designing of ad hoc SCOBY consortia for the targeted synthesis of specific bioactive compounds with recognised utility (biological or technological), thereby broadening applications in food, cosmetic, and pharmaceutical sectors.

## Conclusion

The present work provides new evidence regarding the origin of gallic acid by SCOBY-mediated fermentation of sucrose solutions, outlining a robust framework for further experimentation. The gallic acid content is, to some extent, independent of the phenolic burden provided by the plant material. Thus, it can be modified according to the metabolic traits of the microbial consortia (SCOBY) on chemical constituents of the matrix, for example, sucrose as the carbon source, in the present study. To reach this result, the duration of the fermentation process, as well as the sugar content, directly influences the synthesis of this phenolic acid. Our results further support a relationship between microbial metabolism, the transformation of non-phenolic constituents of fermented beverages, and the degradation, transformation, or synthesis of phenolics. This contrasts with the origin reported upon hydrolysis of the galloyl moiety of esterified flavanols. The evidence gathered in the present work concerning the contribution of bacteria and yeasts to the (poly)phenolic profile of fermented foods will lead to characterising microbial populations with specific metabolic traits. This will provide information that could contribute to obtaining foods with enhanced phytochemical compositions and biological scopes or designing and developing biofactories to produce bioactive compounds usable by a range of industries in the field of health. In connection with the application in the health care sector, suggestions for further studies would include isolating microbial species and characterising their metabolic capacity to synthesise bioactive phenolics.

## Materials and methods

### Chemicals and reagents

The gallic acid standard was acquired from Sigma-Aldrich (Heidelberg, Germany). Acetic and formic acids were obtained from Panreac Lab (Barcelona, Spain). ). The commercial SCOBY was from Kefiralia (Burumart Commerce SL, Guipuzcua, Spain). LC-MS-grade methanol, acetonitrile, and water were purchased from J.T. Baker (Philipsburg, NJ, USA). A Y15 Automatic Analyser was purchased from Biosystems S.A. (Barcelona, Spain). All Y-15 kits for the determination of glucose, fructose, and sucrose (D-Glucose/D-Fructose, ref. 12800 and Sucrose/D-Glucose/D-Fructose, ref. 12819), ethanol (ref. 12847), and acetic acid (ref. 12930), and the corresponding calibrators, controls, system liquids, and washing solutions (refs. 12818, 18063, 12889, and BO131416, respectively) were supplied by BioSystems S.A. (Barcelona, Spain).

### Identification of bacteria in the symbiotic culture

#### DNA extraction

SCOBY was subcultured in a sugared solution (sucrose at 70 g/L) for 14 days at 25 ± 2 °C to obtain enough microbial concentration in the suspension to sequence bacterial DNA. This procedure allowed obtaining a microbial suspension, further used for DNA extraction. Samples (500.0 mL) were centrifuged at 4000 *x rpm* for 20 min, and the supernatant was discarded. The pellet was washed with 5.0 mL of PBS buffer and centrifuged again under matching conditions. The resulting pellet was resuspended in 0.5 mL of PBS buffer and then centrifuged at 12,000 *x g* for 5 min. The supernatant was discarded, and the cell pellet was stored at -80 °C until DNA extraction.

The DNA isolation was carried out with the DNeasy PowerSoil Pro kit (Qiagen, Hilden, Germany) according to the manufacturer’s instructions, with minor modifications described by Peltoniemi et al.^54^. The extracted DNA was purified using NucleoMag^®^ NGS Clean-up and Size Select (Macherey-Nagel, Düren, Germany). For DNA quantification and purity assessment, Qubit^®^ 2.0 Fluorometer (ThermoFisher Scientific, Waltham, Massachusetts, US) and NanoDrop 2000c (ThermoFisher Scientific, Waltham, Massachusetts, US) were used.

#### Amplification and sequencing of bacterial 16 S rRNA gene and bioinformatics analysis

Amplification of bacterial 16 S hypervariable regions was performed on a MiSeq PE300 run (Illumina) at AllGenetics (La Coruña, Spain). Bacterial sequencing analysis was performed with QIIME 2 2020.6^55^ adapted for Illumina data. Raw sequence data were quality-filtered using the q2-demux plugin, followed by denoising with DADA2^56^ (via q2-dada2). Taxonomy was assigned to amplicon sequence variants (ASVs) using the q2-feature-classifier previously described^57^ against the Greengenes 13_8 99% OTU reference sequences.

### Isolation and identification of yeasts in the symbiotic culture

Isolation of yeast colonies from microbial suspension (obtained according to the sub-culture described in the previous subsection) was done by making serial dilutions in plates containing glucose-peptone yeast extract (GPY) consisting of 2.0% glucose, 0.5% peptone, 0.5% yeast extract, and 2.0% bacteriological agar. Plates were cultured for 5–7 days at 25 °C. Several colonies were developed, and 20 of them (randomly chosen) were collected for identification.

The yeasts’ identification was done by sequencing the D1/D2 region of the 26 S rRNA. Genomic DNA was extracted through the lithium acetate (LiOAc)-SDS method^58^. The amplification of the D1/D2 region of the 26 S rRNA gene was achieved using the primers NL1 (5′-GCATATCAATAAGCGGAGGAAAAG-3′) and NL4 (5′-GGTCCGTGTTTCAAGACGG-3′), upon reactions performed in a total volume of 100.0 µL containing 50.0 µL of Nyztech colourless mastermix, 0.5 µL of each primer (10 pmol/µL), 45.0 µL of Milli-Q-H_2_O, and 4.0 µL of DNA. The PCR was carried out in a SimpliAmp PCR system 2700 (Applied Biosystems, Inc., Foster City, CA, USA) by applying 95.0 °C for 3 min, as an initial denaturation condition, followed by 35 cycles of denaturation at 95.0 °C for 30 s, annealing at 55.5 °C for 30 s, elongation at 72.0 °C for 45 s, and a final extension at 72.0 °C for 10 min. The PCR products were run on 1.5% agarose (Pronadisa, Madrid, Spain) gels, stained with real safe, in 0.5x Tris-Borate-EDTA (TBE) buffer. After electrophoresis, gels were visualised under UV light. A 100-bp DNA ladder marker (Roche Molecular Biochemicals, Mannheim, Germany) was used as a size standard.

The amplified DNA was purified in a NucleoFast DNA purification plate (Macherey-Nagel GmbH &Co, Germany) by vacuum in a manifold system (Macherey-Nagel GmbH & Co, Germany). The amplicons were sequenced in both directions, resorting to the BigDye Terminator V3.1 Cycle Sequencing Kit, according to the manufacturer’s instructions (Applied Biosystems, Warrington, UK). The sequencing reactions were run on a Techgene Thermal Cycler (Techne, Cambridge, UK) and involved initial denaturation (94.0 °C, 3 min), followed by 99 cycles of denaturation (96.0 °C, 10 s), annealing (50.0 °C, 5 s), and polymerisation (60.0 °C, 4 min). The nucleotide sequences were obtained with an Applied Biosystems automatic sequencer model ABI-3730 (Applied Biosystems, Warrington, UK). Alignments were done using the Clustal-W algorithm implemented in the MEGA 6.0 software^59^. The aligned sequences were compared to reference data accessible in the GenBank database using the BLASTn tool.

### SCOBY-based model system and fermentation conditions

The SCOBY-based model was developed by adding 70 and 140 g/L sugar into two litres at room temperature (25 ± 2 °C) in four-litre flasks (three biological replicates per condition), without agitation and in darkness, covered by 0.5 mm gauze to get aerobic conditions while avoiding contamination. A commercial SCOBY (first fermentation cycle) (Kefiralia, Burumart Commerce SL, Guipuzcua, Spain) and the starter solution were added to the model solutions (*n* = 3 per experimental condition). Commercial SCOBY was selected to ensure reproducibility and consistency across replicates and experimental conditions, and minimise the interferences that could be associated with home-based SCOBY, thus obtaining a more standardised starting point for the experiments. Two litres of 70 g/L sugar solution without SCOBY constituted the control beverage. Fermentations were conducted in the dark, at 25 ± 2 °C, and under aerobic conditions for 91 days.

For analytical purposes, samples (6 mL) of each independent experimental condition were taken every 7 days until day 91. The collected samples were filtered through 0.22 μm PVDF filters (Millipore, MA, USA). Aliquots were stored at -20 °C until biochemical analyses.

### Analysis of the ethanol, acetic acid, and sugar content

Ethanol and acetic acid were quantified using the Automatic Analyser Y15, following the manufacturer’s instructions. For both of them, the final formation of NADH was measured spectrophotometrically at 340 nm. The concentrations were expressed as mg/L and g/L for ethanol and acetic acid, respectively.

The glucose, fructose, and sucrose concentrations were also determined using the Automatic Analyser Y15. Enzymatic assays were used, and the absorbance of NADPH at 340 nm was measured. The sucrose and its two constituent monosaccharides were expressed as g/L.

### pH and total soluble solids

The pH values were monitored using a GLP 21 pH meter (Crison Ltd., Barcelona, Spain). The total soluble solids (TSS, ºBrix) were recorded using a Pocket Brix-Acidity Meter (Citrus) (PAL-BX/ACID1, Atago Co., Ltd., Tokyo, Japan) at room temperature, according to the manufacturer’s instructions. All samples were analysed on the sampling day (*n* = 3).

### Determination of Gallic acid by UHPLC-ESI-QqQ-MS/MS

For assessing the samples on the concentration of gallic acid, UHPLC-ESI-QqQ-MS/MS-based target metabolomic analyses were applied, according to the methodology described in the literature^60^. Briefly, separation of gallic acid was performed using a UHPLC coupled with a 6460 triple quadrupole-MS/MS (Agilent Technologies, Waldbronn, Germany), using the analytical column BEH C18 1.7 μm (2.1 × 50 mm) (Waters, Milford, MA, USA). The column temperature was set at 30 °C. The mobile phases consisted of deionised Milli-Q water (LC − MS grade)/formic acid (99.9:0.1, *v/v*) (Solvent A) and acetonitrile (Solvent B). The injection volume and flow rate were 15 µL and 0.3 mL/min, respectively. The chromatographic separation of alkyl gallates was achieved upon the following linear gradient (time (min), % B): (0.0, 30%); (3.0, 98%); (6.0, 100%), and (6.1, 30%). An additional post-run period of 1.5 min at 30% solvent B was considered for column equilibration. The identification and quantification of the target analytes were achieved in the negative mode using multiple reaction monitoring (MRM) mass spectrometry, with quantification and confirmation transitions recorded for gallic acid. The MS fragmentor parameters (ion optics: capillary exit voltage) and collision energy were optimised for each analyte. The ionisation and fragmentation conditions were as follows: gas temperature 325 °C, gas flow 8 L/min, nebuliser 30 psi, sheath gas temperature 350 °C, jetstream gas flow 12 L/min, capillary voltage 4000 V, and nozzle voltage 1000 V, according to the most abundant product ions. From the ethanolic stock solutions, successive dilutions at the µM level of concentration were prepared in a 50:50 (*v/v*) mixture of methanol and deionised Milli-Q water to facilitate ionisation in the mass spectrometer. The identification of gallic acid was achieved by comparing retention time (RT) (0.65 min), parent ions (*m/z* [M-H]^−^ at 169 arbitrary mass unit (amu)), and fragmentation product ions (*m/z* MS2[M-H]^−^ at 125 amu (quantification transition), 151 and 71 amu (confirmation transitions)) with the authentic standard. The concentration was calculated by applying standard curves prepared each analysis day and expressed as µg/L. Data acquisition and processing were developed using the MassHunter software version B.08.00 (Agilent Technologies, Waldbronn, Germany).

### Statistical analysis

Experiments were performed in three biological replicates (*n* = 3), and the results were presented as mean ± standard deviation (SD). According to the normal distribution and homogeneity of variance of the data, which were determined by the Shapiro − Wilk (< 50 samples) and Levene tests, correspondingly, one-way analyses of variance (ANOVA) and Tukey’s multiple-range tests were performed using the SPSS v. 28.0 software package (LEAD Technologies, Inc., Chicago, USA). Significant differences were set at *p* < 0.05. A first-order kinetic model for the degradation of sucrose and production of gallic acid by the SCOBY was developed using Excel Professional Plus 2021 (Microsoft, Madrid, Spain).

## Supplementary Information

Below is the link to the electronic supplementary material.

Supplementary Material 1

## Data Availability

The dataset that supports this study’s findings is openly available. The dataset generated and/or analysed during the current study is available in the NCBI Sequence Read Archive (SRA) repository under the BioProject accession numbers PRJNA1249265 ( [16 S rRNA kombucha - SRA - NCBI](https:/www.ncbi.nlm.nih.gov/sra/?term=PRJNA1249265) ) for bacteria and PV583536 and PV583537 ( [*Zygosaccharomyces bailii - NCBI*](https:/www.ncbi.nlm.nih.gov/nuccore/2966924252) and [*Rhodotorula mucilaginosa - NCBI*](https:/www.ncbi.nlm.nih.gov/nuccore/2966924253) , respectively) for yeasts.
