# Supplementary material for: SCOBY-based, innovative, and sustainable production of gallic acid from sucrose towards multipurpose applications
Source: Sci Rep. 2025 Nov 18;15:40536. doi: 10.1038/s41598-025-24371-3 (PMC12627677; doi:10.1038/s41598-025-24371-3)

SUPPLEMENTARY MATERIAL

| **Table S1.** Summary table displaying key fermentation parameters at 70 g sucrose/L. | | | | |
| --- | --- | --- | --- | --- |
| ***Microbial profile*** | | | | |
| **Genera** | | | **Percentage** | |
| Bacteria | | | | |
| *Komagateibacter* | | >99.79 % | | |
| *Acinetobacter* | | 0.09 % | | |
| *Enterobacter* | | 0.05 % | | |
| *Burkholderia* | | 0.03 % | | |
| *Pseudomonas* | | 0.02 % | | |
| *Exiguobacterium* | | ≤0.1 % | | |
| *Rhizobiaceae* | | ≤0.1 % | | |
| *Sphingobium* | | ≤0.1 % | | |
| *Bosea* | | ≤0.1 % | | |
| Yeast | | | | |
| *Zygosaccharomyces bailii* | | N.d. | | |
| *Rhodotorula mucilaginosa* | | N.d. | | |
|  | | | | |
| ***Fermentation parameters*** | | | | |
|  | *t_0_* | |  | *t_91_* |
| Sucrose (g/L) | 71.41 | |  | 22.02 |
| *k* (day^–1^) | --- | |  | 0.011 |
| Gallic acid (µg/L) | 0.00 | |  | 62.91 |
| Conversion rate (%) | --- | |  | 63.2 |
| pH | 7.72 | |  | 3.04 |
| Ethanol (mg/L) | 0.00 | |  | 250.50 |
| Acetic acid (g/L) | 0.00 | |  | 2.11 |
| N.d., not determined. | | | | |

**Figure S1.** First-order kinetic model for degradation of sucrose (at 70 g/L) and production of gallic acid by a Symbiotic Culture of Bacteria and Yeast (SCOBY) during 91 days, at 25 ± 2 ºC.


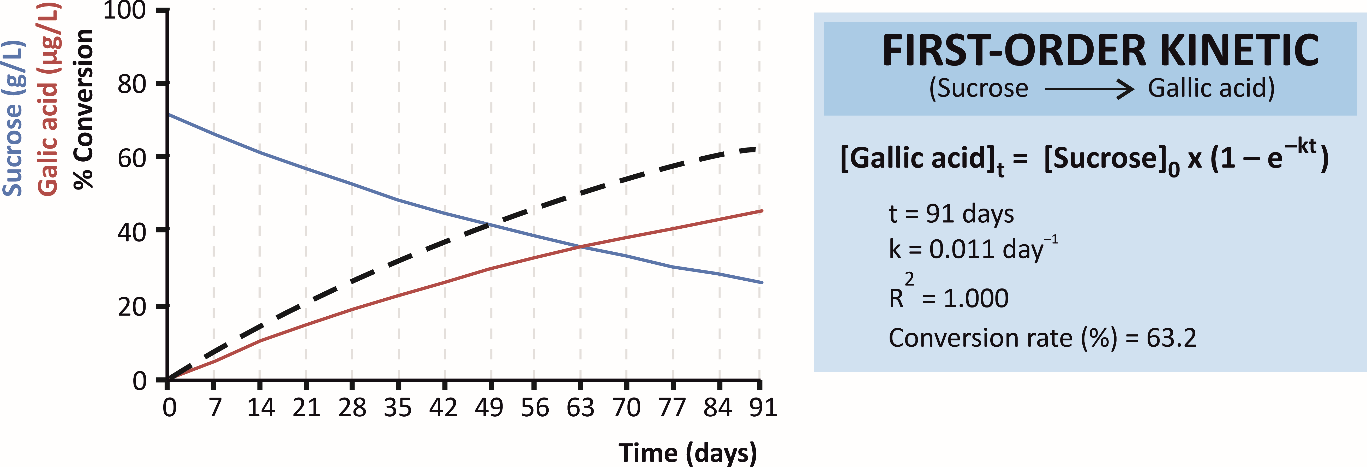

Supplement: Supplementary file 1 — Supplementary Material 1 [file 41598_2025_24371_MOESM1_ESM.docx]
